# Influence of contrast media on renal function and outcomes in patients with sepsis-associated acute kidney injury: a propensity-matched cohort study

**DOI:** 10.1186/s13054-019-2517-3

**Published:** 2019-07-09

**Authors:** Yuya Goto, Kansuke Koyama, Shinshu Katayama, Ken Tonai, Jun Shima, Toshitaka Koinuma, Shin Nunomiya

**Affiliations:** Division of Intensive Care, Department of Anesthesiology & Intensive Care Medicine, Jichi Medical University School of Medicine, 3311-1 Yakushiji, Shimotsuke, Tochigi 329-0498 Japan

**Keywords:** Post-contrast acute kidney injury, Sepsis-associated AKI, Contrast media, Critical care

## Abstract

**Background:**

Recent studies have suggested a low potential risk for contrast medium-induced kidney injury in patients with relatively normal renal function. However, whether contrast media cause additional deterioration of renal function in patients with acute kidney injury (AKI), including those with sepsis-associated AKI, remains unclear. This study aimed to evaluate the effect of contrast media on renal function and mortality in patients with sepsis who already had AKI.

**Methods:**

We performed a propensity score-matched historical cohort study in the medico-surgical intensive care unit of Jichi Medical University Hospital. Adult patients who were diagnosed with sepsis and AKI were enrolled. Records from our sepsis database from 2011 to 2017 were examined. Septic patients with AKI who received contrast media within 24 h of admission (C group) were matched 1:1 with septic patients who did not receive contrast media (NC group). The primary outcome was deterioration of kidney function (DRF), which was defined as an elevation of serum creatinine levels (> 0.3 mg/dL or 1.5-fold from baseline) or induction of renal replacement therapy.

**Results:**

A total of 339 septic patients with AKI were included. After propensity score adjustment, the DRF rate was similar between the C and NC groups (34.0% versus 35.0%; *P* = 1.00). The 7-day mortality (3.0% versus 6.0%; *P* = 0.50), 28-day mortality (9.2% versus 15.0%; *P* = 0.25), and 90-day mortality (25.8% versus 32.1%; *P* = 0.45) rates were comparable between the two groups. In propensity-adjusted subsets of a high-risk subset (AKI stages 2 and 3 on admission), the rate of DRF was also similar between the two groups.

**Conclusions:**

A single administration of contrast media was not associated with exacerbation of AKI or increased short/long-term mortality in patients with sepsis.

**Electronic supplementary material:**

The online version of this article (10.1186/s13054-019-2517-3) contains supplementary material, which is available to authorized users.

## Background

Contrast media (CM) have long been regarded as a potential risk for nephropathy. Multiple risk factors, including diabetes, sepsis, anemia, hypotension, circulatory insufficiency, and nephrotoxic agents, can increase the risk of contrast-associated acute kidney injury (CA-AKI) [1–5]. However, the majority of evidence for contrast nephrotoxicity is from cardiology procedures, such as coronary angiography and/or intervention. Performing a controlled study in this situation is difficult because the procedures always require CM. In the critical care setting, several studies have reported that CA-AKI occurs in 11–23% [1–3, 6–8] of patients who undergo a radiological examination and is associated with a worse outcome. However, those studies only examined patients who were exposed to CM and did not exclude potential causes other than CM.

Recently, the evidence for CA-AKI has been challenged in several studies, including a meta-analysis, which compared critically ill patients who were scanned with or without radiocontrast enhancement. In these studies, CM exposure was not associated with decreased renal function or an increased incidence of nephropathy in critically ill patients [9–11]. However, the effects of CM in septic patients, especially those who already have AKI, remain unknown. Patients with AKI are often considered vulnerable to CM nephrotoxicity. Determining whether CM induces further deterioration of renal function is clinically important because physicians often face the dilemma between reliable diagnosis of the infectious source and exacerbation of septic AKI when considering contrast-enhanced examinations. Additionally, whether AKI is the same as chronic kidney disease as a risk factor for contrast nephropathy is undetermined. AKI is a disorder of the proximal tubules, and chronic kidney disease is histologically different from AKI, with nephron injury and fibrosis. Therefore, the risk of AKI for development of CM toxicity for renal function needs to be determined.

The present study aimed to examine the effect of CM on renal function in patients with sepsis who already had AKI. We hypothesize that CM exposure is not associated with deterioration of renal function (DRF) upon sepsis-associated AKI, which was defined as an elevation of serum creatinine (Cr) levels or induction of renal replacement therapy (RRT). We conducted a propensity-matched historical cohort study to investigate the additional DRF and mortality in septic patients with AKI depending on CM exposure.

## Methods

### Study design and setting

This was a single-center, retrospective, propensity-matched cohort study in a 14-bed medico-surgical intensive care unit (ICU) of Jichi Medical University Hospital. The database of all patients who were admitted to the ICU from June 2011 to December 2017 was reviewed. Patients ≥ 18 years of age who were diagnosed with sepsis and AKI on admission to the ICU were included in the study. The diagnosis of sepsis was based on the Sepsis-3 criteria [12]. We defined AKI on the basis of serum Cr criteria of Kidney Disease Improving Global Outcomes (KDIGO) guidelines [13] (i.e., an absolute increase in 0.3 mg/dL or a 50% increase from baseline on admission). Baseline Cr was defined as premorbid Cr if available or estimated creatinine obtained by solving the four-variable Modification of Diet in Renal Disease equation for a low normal glomerular filtration rate (GFR) of 75 mL/min/1.73 m^2^ [14]. Premorbid serum Cr was defined as the last available measurement from 365 days to 7 days before hospital admission based on a previous report [15].

Exclusion criteria were a history of renal transplantation, ongoing or previous hemodialysis, insufficient data of urine output or Cr, and death within 24 h of admission. We also excluded patients with exposure of CM 2–3 days before admission or patients who underwent two or more examinations of contrast-enhanced computed tomography (CT) scans or multiple procedures using CM within the first week of admission. Eligible patients were divided into the following two groups on the basis of exposure of CM: patients who underwent a single examination or procedure using CM at the time or within 24 h before ICU admission (C group) and patients without CM exposure (NC group). We also stratified eligible patients into a high-risk subset (AKI stages 2 and 3 at ICU admission) and low-risk subset (AKI stage 1 at ICU admission) and separately performed propensity score analysis. This study was approved by the Institutional Research Ethics Committee of Jichi Medical University.

### Data collection

We collected the following variables. Patients’ baseline characteristics included age, sex, Acute Physiology and Chronic Health Evaluation (APACHE) II score, Sequential Organ Failure Assessment score, height, weight, history of diabetes mellitus, ischemic heart disease, chronic kidney disease, infection site, presence of septic shock and disseminated intravascular coagulation, and data on administration of aminoglycoside or vancomycin. Patients’ outcomes included the requirement for RRT within 7 days after admittance into the ICU, ventilator-free days, ICU-free days, persistent requirement for renal support during the general ward stay or after hospital discharge, and mortality rate.

### Outcome measures

The primary outcome was DRF, which was defined by an increase in serum Cr levels ≥ 0.3 mg/dL or 1.5-fold from serum Cr level measurement on admission or induction of RRT within 72 h of ICU admission (administration of CM in the C group). Elevation of serum Cr levels was determined by reference to the KDIGO criteria [13, 16]. The incidence of CA-AKI can differ depending on the definition used [1–3, 6–8, 17]. Therefore, we analyzed patients’ outcomes using an alternative definition (i.e., the Barrett and Parfrey criteria) [18]. The secondary outcomes were ventilator-free days, ICU-free days, and short-term (7-day, 28-day) and long-term (90-day) mortality rates. We also investigated the use of temporal or chronic hemodialysis after discharge from the ICU and our hospital. We categorized patients into three groups on the basis of those who did not require hemodialysis after ICU discharge, those who required hemodialysis during general ward hospitalization, and those who required chronic hemodialysis after hospital discharge.

### Statistical analysis

Variables were compared between the two groups using Fisher’s exact test, Pearson’s chi-square test, and the Mann-Whitney *U* test as appropriate. Because administration of CM was not randomized, we generated a propensity score by logistic regression between patients who received CM and those who did not. Propensity score covariate adjustment was selected as the analysis technique for explaining the factors that affected the use of CM or factors related to the prognosis or renal function of the patients. The variables of age, sex, infection site, history of chronic kidney disease, diabetes mellitus, septic shock, immune compromise, use of aminoglycoside or vancomycin, laboratory data (hemoglobin, Cr, total bilirubin, platelets, lactate), the ratio of partial pressure of oxygen to the fraction of inspired oxygen, and the Glasgow Coma Scale on ICU admission were used to create the propensity score. We also generated the propensity-matched contrast and non-contrast groups for each low- and high-risk subset. Risk factors for DRF in patients with AKI on admission were assessed using multivariate logistic regression, including clinically plausible variables associated with use of CM or exacerbation of renal function (i.e., age, sex, infection site, history of CKD, APACHE II scores, septic shock, use of aminoglycoside or vancomycin, disseminated intravascular coagulation, serum Cr on admission, and administration of CM). All statistical analyses were performed with statistical software (JMP 13; SAS Institute Inc., Cary, NC, USA). Unless specified, data are reported as median (25th–75th percentile), mean (± standard deviation), or count (%). *P* values < 0.05 were considered statistically significant.

## Results

**Fig. 1:**
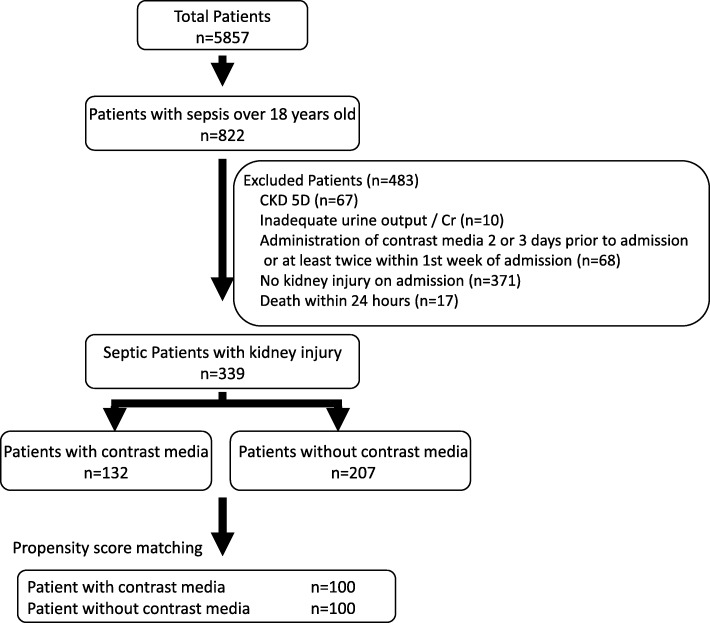
Study inclusion flow chart. CKD, chronic kidney disease; AKI, acute kidney injury

Of 5857 patients who were admitted to our ICU during the study period, 822 were diagnosed with sepsis, while 483 were excluded according to our exclusion criteria. The remaining 339 patients were included in our study. Of these, 136 (40.1%) patients received CM on the day of admission or within 24 h before admission (Fig. 1). All administration of CM was used for CT scan imaging. No patients used CM for intervention. The characteristics of the unadjusted and propensity score-matched patients are shown in Table 1. Before propensity score matching, patients in the C group had more abdominal or neck infection (62.1% versus 45.9%, *P* < 0.01, and 9.1% versus 0.5%, *P* < 0.01, respectively), less thoracic and urinary tract infection (10.6% versus 26.1%, *P* < 0.01, and 1.5% versus 8.2%, *P* = 0.01, respectively), and a lower renal Sequential Organ Failure Assessment score (1 (0–1) versus 1 (1–2), *P* < 0.01) compared with the NC group. Patients in the C group also had lower serum levels of Cr (1.38 mg/dL versus 1.91 mg/dL, *P* < 0.01), blood urea nitrogen (31.0 mg/dL versus 44.0 mg/dL, *P* < 0.01), urinary acid (5.4 mg/dL versus 6.4 mg/dL, *P* = 0.01), and cystatin C (1.58 mg/dL versus 2.01 mg/dL, *P* < 0.01) and higher hemoglobin (10.5 g/dL versus 9.5 g/dL, *P* < 0.01) before matching. After propensity score matching, 100 pairs of patients were selected, and almost all significant differences in the patients’ characteristics and laboratory data between the two groups were eliminated.

**Table 1:** Patients’ characteristics and laboratory data on admission in the unmatched (a) and matched groups (b)

(a)	C group (*n* = 132)	NC group (*n* = 207)	*P* value
Age, years (IQR)	70.5 (60–78)	71 (61–78)	0.38
Male, *n* (%)	70 (54.4)	124 (58.8)	0.32
Site of infection, *n* (%)			*< 0.01*
Central nervous system	1 (0.8)	0 (0.0)	0.39
Thoracic, pneumonia	14 (10.6)	54 (26.1)	*< 0.01*
Abdominal	82 (62.1)	95 (45.9)	*< 0.01*
Neck	12 (9.1)	1 (0.5)	*< 0.01*
Soft tissue	8 (6.1)	10 (4.8)	0.62
Urinary tract	2 (1.5)	17 (8.2)	*0.01*
CRBSI	0 (0.0)	4 (1.9)	0.16
Others	13 (9.9)	26 (12.6)	0.37
Nephrotoxic agents, *n* (%)			
Aminoglycoside	1 (0.8)	3 (1.5)	1.00
Vancomycin	42 (31.8)	60 (29.0)	0.58
Comorbidities, *n* (%)			
Ischemic heart disease	17 (12.9)	21 (10.1)	0.46
Chronic kidney disease	17 (12.9)	39 (18.8)	0.15
Premorbid Cr (mg/dL) (IQR)	0.85 (0.561–0.97), *n* = 60	0.8 (0.51–1.08), *n* = 128	0.12
eGFR < 30 (%)	3 (5.0)	20 (15.6)	0.05
Diabetes mellitus	35 (26.5)	63 (30.4)	0.44
Immunosuppressants	28 (21.2)	72 (34.8)	*0.01*
APACHE II score (IQR)	24 (18–30)	26 (20–32)	0.17
SOFA score (IQR)	8 (6–10.8)	9 (6–11)	0.14
SOFA (non-renal SOFA)	7 (5–9)	7 (5–9)	0.89
SOFA (renal SOFA)	1 (0–1)	1 (1–2)	*< 0.01*
DIC, *n* (%)	69 (53.5)	12 (60.81)	0.19
Septic shock, *n* (%)	72 (54.6)	104 (50.2)	0.44
AKI stage on admission, *n* (%)			*< 0.01*
Stage 1	63 (47.7)	55 (26.6)	
Stage 2	37 (28.0)	49 (23.7)	
Stage 3	32 (24.2)	103 (49.8)	
Lab data on admission			
WBC (10^9^ cells/L) (IQR)	10.4 (4.8–16.6)	9.5 (4.8–14.9)	0.17
Hb (g/dL) (IQR)	10.5 (9.1–12.4)	9.8 (8.4–11.5)	*< 0.01*
Platelet (10^4^ cells/μL) (IQR)	14.1 (8.6–20.4)	12.7 (7.4–18.4)	0.67
AT III (%) (IQR)	51.5 (38.9–62.4)	52.1 (41.9–63.2)	0.46
Protein C (%) (IQR)	45.0 (33.2–66.5)	47.1 (34.7–60.2)	0.74
Albumin (mg/dL) (IQR)	2.3 (2–2.8)	2.3 (1.9–2.8)	0.14
Bilirubin (mg/dL) (IQR)	1.12 (0.63–1.89)	0.81 (0.58–1.46)	0.52
PCT (ng/mL) (IQR)	11.4 (2.0–63.7)	13.5 (3.3–63.9)	0.77
CRP (mg/dL) (IQR)	13.4 (6.8–24.4)	15.4 (8.1–25.0)	0.51
Cr (mg/dL) (IQR)	1.38 (1.01–1.96)	1.91 (1.33–2.98)	*< 0.01*
BUN (mg/dL) (IQR)	31.0 (21.3–41.8)	44.0 (28.0–65.0)	*< 0.01*
UA (mg/dL) (IQR)	5.4 (4.3–6.9)	6.4 (5.1–8.5)	*< 0.01*
Cystatin C (mg/L) (IQR)	1.58 (1.21–2.12)	2.01 (1.46–2.84)	*0.01*
BNP (pg/mL) (IQR)	172.5 (72.3–546.1)	208.3 (65.4–538.5)	0.68
Lactate (mmol/L) (IQR)	2.6 (1.5–4.6)	2.4 (1.5–4.1)	0.20
(b)	C group (*n* = 100)	NC group (*n* = 100)	*P* value
Age, years (IQR)	70.5 (61–79.8)	71 (60–79)	0.95
Male, *n* (%)	52 (52.0)	57 (57.0)	0.48
Site of infection, *n* (%)			0.86
Central nervous system	0 (0.00)	0 (0.00)	
Thoracic, pneumonia	13 (13.0)	12 (12.0)	0.83
Abdominal	66 (66.0)	70 (70.0)	0.54
Neck	1 (1.0)	1 (1.0)	1.00
Soft tissue	8 (8.0)	7 (7.0)	0.79
Urinary tract	2 (2.0)	4 (4.0)	0.68
CRBSI	0 (0.0)	0 (0.0)	
Others	10 (10.0)	7 (7.0)	0.45
Nephrotoxic agents, *n* (%)			
Aminoglycoside	1 (1.0)	0 (0.0)	0.32
Vancomycin	33 (33.0)	32 (32.0)	0.88
Comorbidities, *n* (%)			
Ischemic heart disease	14 (14.0)	9 (9.0)	0.27
Chronic kidney disease	14 (14.0)	14 (14.0)	1.00
Premorbid Cr (mg/dL) (IQR)	0.85 (0.62–0.96), *n* = 50	0.76 (0.57–0.92), *n* = 48	0.20
eGFR < 30 (%)	1 (2.0)	1 (2.1)	0.98
Diabetes mellitus	26 (26.0)	28 (28.0)	0.75
Immunosuppressants	25 (25.0)	22 (22.0)	0.62
APACHE II score (IQR)	24 (19–30)	23 (18–29)	0.20
SOFA score (IQR)	8 (6–11)	8 (5.3–10)	0.97
SOFA (non-renal SOFA)	7 (5–9.8)	7 (4–9)	0.84
SOFA (renal SOFA)	1 (0–2)	1 (1–2)	0.60
DIC, *n* (%)	55 (55.0)	57 (57.0)	0.90
Septic shock, *n* (%)	54 (54.0)	59 (59.0)	0.48
AKI stage on admission, *n* (%)			0.20
Stage 1	44 (44.0)	32 (32.0)	
Stage 2	26 (26.0)	29 (29.0)	
Stage 3	30 (30.0)	39 (39.0)	
Lab data on admission			
WBC (10^9^cells/L) (IQR)	10.9 (4.4–15.5)	8.7 (4.8–14.8)	0.18
Hb (g/dL) (IQR)	10.4 (9.0–12.0)	10.9 (9.2–12.0)	0.36
Platelet (10^4^cells/μL) (IQR)	13.0 (7.4–20.6)	12.8 (7.7–17.8)	0.74
AT (%) (IQR)	50.1 (37.8–60.1)	49.1 (39.2–61.1)	0.82
Protein C (%) (IQR)	44.5 (29.9–61.6)	43.2 (32.3–56.8)	0.66
Albumin (mg/dL) (IQR)	2.3 (2.0–2.8)	2.5 (1.9–2.9)	0.99
Bilirubin (mg/dL) (IQR)	1.04 (0.62–1.86)	0.91 (0.66–1.61)	0.78
PCT (ng/mL) (IQR)	13.1 (2.2–76.7)	25.5 (5.3–86.9)	0.57
CRP (mg/dL) (IQR)	13.0 (7.2–24.4)	14.4 (8.0–25.0)	0.63
Cr (mg/dL) (IQR)	1.47 (1.03–2.20)	1.63 (1.20–2.32)	0.90
BUN (mg/dL) (IQR)	33.5 (24.3–43.8)	38.0 (25–51.0)	0.61
UA (mg/dL) (IQR)	5.7 (4.4–7.0)	6.1 (4.8–7.3)	0.75
Cystatin C (mg/L) (IQR)	1.68 (1.28–2.27)	1.62 (1.23–2.13)	0.14
BNP (pg/mL) (IQR)	184 (73.2–559)	144.9 (55.6–427.7)	0.06
Lactate (mmol/L) (IQR)	2.4 (1.5–4.9)	2.6 (1.7–4.1)	0.49

Italicized *P* values represent significant differences between the C and NC groups

*Abbreviations*: *CRBSI* catheter-related bloodstream infection, *APACHE II score* Acute Physiology and Chronic Health Evaluation II score, *SOFA score* Sequential Organ Failure Assessment score, *DIC* disseminated intravascular coagulation, *AKI* acute kidney injury, *WBC* white blood cell, *Hb* hemoglobin, *AT* antithrombin, *PCT* procalcitonin, *CRP* C-reactive protein, *UA* urinary acid, *Cr* creatinine, *BUN* blood urea nitrogen, *BNP* brain natriuretic peptide, *IQR* interquartile range

### Outcomes after propensity score matching

In unadjusted and propensity-matched analyses, the rate of DRF was comparable between the C and NC groups (34.1% versus 43.0% in unadjusted, 34.0% versus 35.0% in matched groups, Table 2). In the propensity score-matched groups, there was no difference in ICU-free days, ventilator-free days, or mortality between the two groups. Only a few patients required chronic hemodialysis after hospital discharge in both groups (C group versus NC group, 0% versus 2.0%, not significant). The outcomes for propensity score-adjusted subsets of high- and low-risk subsets are shown in Tables 3 and 4, respectively. The rate of DRF was similar between the groups, and there were no differences in other outcomes.

**Table 2:** Outcomes of the unmatched (a) and matched (b) groups

(a)	C group (*n* = 132)	NC group (*n* = 207)	*P* value
Deterioration of renal function, *n* (%)	45 (34.1)	89 (43.0)	0.10
Cr elevation	24 (18.2)	40 (19.3)	0.79
RRT induction	31 (23.5)	69 (33.3)	0.05
RRT after ICU discharge, *n* (%)	11 (8.1)	25 (11.9)	0.29
Chronic HD after hospital discharge, *n* (%)	0 (0.0)	7 (3.3)	*0.05*
Ventilator-free days (mean, SD)	16.2 ± 9.9	16.4 ± 10.6	0.85
ICU-free days (mean, SD)	16.7 ± 7.5	16.4 ± 8.2	0.74
7-day mortality, *n* (%)	4 (3.0)	13 (6.3)	0.21
28-day mortality, *n* (%)	9 (7.8)	29 (16.4)	*0.03*
90-day mortality, *n* (%)	19 (21.8)	43 (35.0)	*0.04*
(b)	C group (*n* = 100)	NC group (*n* = 100)	*P* value
Deterioration of renal function, *n* (%)	34 (34.0)	35 (35.0)	1.00
Cr elevation	15 (15.0)	19 (19.0)	0.57
RRT induction	26 (26.0)	23 (23.0)	0.74
RRT after ICU discharge, *n* (%)	7 (7.0.)	6 (6.0)	0.77
Chronic HD after hospital discharge, *n* (%)	0 (0.0)	2 (2.0)	0.50
Ventilator-free days (mean, SD)	16.0 ± 10.0	16.3 ± 10.8	0.84
ICU-free days (mean, SD)	16.9 ± 7.4	17.8 ± 7.6	0.38
7-day mortality, *n* (%)	3 (3.0)	6 (6.0)	0.50
28-day mortality, *n* (%)	8 (9.2)	12 (15.0)	0.25
90-day mortality, *n* (%)	17 (25.8)	17 (32.1)	0.45

Italicized P values represent significant differences between the C and NC groups

*Abbreviation*: *RRT* renal replacement therapy, *ICU* intensive care unit, *HD* hemodialysis, *C group* patients who received contrast media, *NC group* patients who did not receive contrast media, *SD* standard deviation

**Table 3:** Outcomes of the matched groups (AKI stage 1 on admission)

	C group (*n* = 22)	NC group (*n* = 22)	*P* value
Deterioration of renal function, *n* (%)	4 (18.2)	6 (27.3)	0.72
Cr elevation	3 (13.6)	5 (22.7)	0.70
RRT induction	1 (4.6)	1 (4.6)	1.00
RRT after ICU discharge, *n* (%)	0 (0.0)	0 (0.0)	
RRT after hospital discharge (Chronic HD), *n* (%)	0 (0.0)	0 (0.0)	
Ventilator-free days (mean, SD)	19.3 ± 5.8	18.0 ± 10.7	0.53
ICU-free days (mean, SD)	16.5 ± 6.6	12.1 ± 10.7	0.11
7 days mortality, *n* (%)	0 (0.0)	0 (0.0)	
28 days mortality, *n* (%)	0 (0.0)	3 (18.8)	0.08
90 days mortality, *n* (%)	2 (14.3)	3 (25.0)	0.64

*Abbreviation*: *AKI* acute kidney injury, *Cr* creatinine, *RRT* renal replacement therapy, *ICU* intensive care unit, *HD* hemodialysis, *C group* patients who received contrast media, *NC group* patients who did not receive contrast media, *SD* standard deviation

**Table 4:** Outcomes of the matched groups (AKI stages 2 and 3 on admission)

	C group (*n* = 53)	NC group (*n* = 53)	*P* value
Deterioration of renal function, *n* (%)	23 (43.4)	20 (37.7)	0.55
Cr elevation	11 (20.8)	10 (18.9)	0.81
RRT induction	20 (37.7)	16 (30.2)	0.41
RRT after ICU discharge, *n* (%)	8 (15.1)	4 (7.6)	0.36
RRT after hospital discharge (Chronic HD), *n* (%)	0 (0.0)	2 (3.3)	0.50
Ventilator-free days (mean, SD)	14.2 ± 9.9	14.9 ± 10.9	0.75
ICU-free days (mean, SD)	14.7 ± 8.0	16.2 ± 8.5	0.38
7 days mortality, *n* (%)	3 (5.6)	6 (11.3)	0.48
28 days mortality, *n* (%)	7 (14.0)	10 (21.3)	0.35
90 days mortality, *n* (%)	12 (31.6)	13 (41.9)	0.37

*Abbreviation*: *AKI* acute kidney injury, *Cr* creatinine, *RRT* renal replacement therapy, *ICU* intensive care unit, *HD* hemodialysis, *C group* patients who received contrast media, *NC group* patients who did not receive contrast media, *SD* standard deviation

### Risk factors for deterioration of renal function in patients with AKI

To identify the risk factors for DRF and assess the independent association between CM and DRF, we divided the study cohort into patients with DRF and without DRF (Additional file 1: Table S1) and performed multivariable regression analysis including clinically plausible variables related to the use of CM or exacerbation of renal function. The multivariate logistic regression revealed CKD (odds ratio (OR), 3.16; 95%CI, 1.49–6.72), APACHE II score (OR, 1.15; 95%CI, 1.10–1.20), DIC (OR, 1.84; 95%CI, 1.04–3.25), and admission creatinine (OR, 1.32; 95%CI, 1.11–1.60) as independent risk factors for DRF. However, administration of CM was not associated with DRF in the multivariate regression models (OR, 0.94; 95%CI, 0.53–1.69, Table 5).

**Table 5:** Multivariate logistic regression analysis assessing the risk factors for deterioration of renal function in sepsis patients with AKI

Factors	OR	95% CI	*P* value
Age	0.98	0.97–1.00	0.12
Sex, male	1.71	0.98–2.96	0.06
Aminoglycoside	4.78	0.39–59.4	0.22
Vancomycin	1.63	0.89–2.99	0.11
CKD	3.16	1.49–6.70	*< 0.01*
Septic shock	1.09	0.60–1.95	0.78
DIC	1.84	1.04–3.25	*0.04*
Administration of CM	0.94	0.53–1.69	0.84
Infection site-thoracic	1.65	0.73–3.73	0.23
Infection site-abdominal	1.88	0.93–3.81	0.08
Cr	1.32	1.11–1.60	*< 0.01*
APACHE II score	1.15	1.10–1.20	*< 0.01*

Italicized P values represent significant differences

*Abbreviations*: *OR* odds ratio, *CI* confidence interval, *CKD* chronic kidney disease, *DIC* disseminated intravascular coagulation, *CM* contrast media, *Cr* creatinine, *APACHE II score* Acute Physiology and Chronic Health Evaluation II score

## Discussion

In the present study, we investigated the influence of CM administration on the outcomes in septic patients with AKI in the ICU. We found that there was no significant further DRF and no additional increase in the induction of RRT in patients with AKI who received CM compared with those who did not receive CM. Further, these results were robust, even in the subgroup of patients with severe renal impairment (AKI stages 2 and 3).

Several recent studies examined the relationship between CM and development of renal impairment in critically ill patients and suggested that the incidence of CM-induced nephropathy was low [4, 19, 20]. Cely et al. [4] reported that decreased renal function was common following contrast-enhanced CT scanning, but that the changes were mostly caused by factors other than contrast exposure itself. Further, in a meta-analysis, Ehrmann et al. [11] found no evidence for the development of AKI attributable to CM in critically ill patients. Our results are relatively similar to these findings, although these previous studies enrolled patients with stable renal function at baseline, while our study patients had already developed AKI before CM exposure. Additionally, we focused on septic patients, who are considered as high risk for decreased renal function. The proportion of patients with sepsis in a recent large study was approximately 30%, which may be insufficient to examine the effect of CM administration in septic patients [10]. Our results provide further support for low CM toxicity in septic patients with AKI. This suggests that withholding CM-enhanced imaging studies may not be necessary when an accurate diagnosis is required, even in patients with decreasing renal function.

There are two main reasons why CM administration was not associated with decreased renal function or a poor prognosis in patients with sepsis-associated AKI. First, the specific additional toxicity of CM may be low [11, 21]. Second, septic AKI has a high recovery rate of renal function. Although septic AKI is strongly associated with worse outcomes, complete recovery of renal function occurs in > 90% of septic patients with AKI, with a mean time for recovery of approximately 10 days [22]. Indeed, 98.0% of patients in the NC group and 100% in the C group were free from chronic hemodialysis at hospital discharge in our study.

The primary outcome of our study was the rate of DRF. We defined DRF as elevated Cr levels and induction of RRT. Because there is no widely accepted definition for the Cr threshold that indicates DRF, we defined the threshold on the basis of the KDIGO criteria [16]. We also analyzed the same data using the Barrett and Parfrey criteria [18] and found no differences in Cr elevation with either diagnostic criteria (data not shown). There was no difference in the RRT induction rate between the two groups in the present study. The rate of RRT introduction in septic AKI was reported as 24–89% [23, 24]. Further, the rate of induction of hemodialysis in the NC group was 26% in the present study, which is similar to that previously reported [23, 24], and suggests no additional effect of the use of CM. We also investigated the short-term and long-term mortality rates as secondary outcomes. Although there were no significant differences in short-term and long-term mortality rates between the two groups, the mortality rates at 28 and 90 days were higher in the NC group compared with the C group.

In clinical practice, physicians are often faced with the difficult decision on whether to use CM for enhanced imaging to evaluate the infectious source in septic patients with severe renal dysfunction. We found no significant effects of CM on major outcomes in a subgroup of patients with AKI stages 2 and 3. These results contrast with the findings of McDonald et al., who found that an increased risk of dialysis was observed in the contrast group patients with an estimated GFR < 45 mL/min/1.73 m^2^ [10]. However, in their study, there was no significant difference in the rate of AKI development between the contrast and non-contrast groups, although the non-contrast group had a greater decrease in serum Cr levels after an unenhanced CT scan. Additionally, our study focused on patients who already had AKI. Therefore, the effects of CM on the changes in Cr levels or induction of RRT would be different between patients with AKI and patients with chronic kidney disease. Future large multicenter studies are required to evaluate this higher risk population with renal insufficiency.

Our study has several limitations. First, because our study was retrospective from a single ICU, the population size was relatively small. There may have been selection bias, and our results may not be generalizable. Nevertheless, prospective studies on the use of CM are ethically difficult [9, 10]. Further multicenter studies on the effect of CM administration in septic patients with AKI may be warranted. Second, propensity score adjustment can only account for measured or known confounders. While our model included 16 clinical covariates, unmeasured confounders may still remain in our study that could affect the patient outcomes. Third, patients in the NC group did not always receive a CT examination. Patients requiring CT may be sicker than other patients [20]. Furthermore, the reasons why CM was not used in patients in the NC group are unknown, although this may relate to confounding factors that were not identified, which may have affected our results. Fourth, our AKI phase data are missing, although we targeted patients with impaired renal function at entry. Therefore, whether AKI was in the recovery or deterioration period is unknown. However, considering that the patients’ background and severity between the two groups were consistent with the propensity score, the phase of AKI was unlikely to be significantly different between the two groups. Fifth, all administration of CM was used for CT scan imaging. No patients received CM for intervention. Our results cannot be adopted for the use of CM for interventional purposes in septic patients with AKI. Additionally, the dose of CM per patient was not available in this study. The use of > 100 mL of contrast is associated with an increased risk of kidney injury [25]. The effect of contrast-enhanced CT using a large amount of CM is unknown. Sixth, we did not have sufficient information on other drugs that may affect renal function, except for vancomycin and aminoglycoside. Finally, because CM administration was mainly performed for examining the infectious source, the prognosis may have been affected by earlier diagnosis in patients with CM. However, the comparison between the groups that used propensity score matching showed no correlation with a poor prognosis in the C group. The reason for withholding the use of CM for accurate diagnosis in our patients remains unclear.

## Conclusions

A single administration of CM for a CT scan was not associated with further deterioration of renal function in patients with sepsis-associated AKI. Further, even in patients with a high-risk subset, there was no evidence of adverse effects on renal function or short-term and long-term mortality. Our results suggest that contrast-enhanced imaging should not be avoided in clinical practice if required to evaluate the source of infection.

## Additional file

Additional file 1: **Table S1.** Comparison of the study patients with and without DRF. (DOCX 18 kb)

## Data Availability

The datasets used during the current study are available from the corresponding author on request.

## Abbreviations

AKI: Acute kidney injury
APACHE II score: Acute Physiology and Chronic Health Evaluation II score
AT: Antithrombin
BNP: Brain natriuretic peptide
BUN: Blood urea nitrogen
CA-AKI: Contrast-associated acute kidney injury
CI: Confidence interval
CKD: Chronic kidney disease
Cr: Creatinine
CRBSI: Catheter-related bloodstream infection
CRP: C-reactive protein
CT: Computed tomography
DIC: Disseminated intravascular coagulation
DRF: Deterioration of renal function
GFR: Glomerular filtration rate
Hb: Hemoglobin
HD: Hemodialysis
ICU: Intensive care unit
IQR: Interquartile range
KDIGO: Kidney Disease Improving Global Outcomes
OR: Odds ratio
PCT: Procalcitonin
RRT: Renal replacement therapy
SD: Standard deviation
SOFA score: Sequential Organ Failure Assessment score
UA: Urinary acid
WBC: White blood cell
