# Supplementary material for: Influence of contrast media on renal function and outcomes in patients with sepsis-associated acute kidney injury: a propensity-matched cohort study
Source: Crit Care. 2019 Jul 9;23:249. doi: 10.1186/s13054-019-2517-3 (PMC6615173; doi:10.1186/s13054-019-2517-3)
Supplement: Supplementary file 1 — Table S1. Comparison of the study patients with and without DRF. (DOCX 18 kb) [file 13054_2019_2517_MOESM1_ESM.docx]

Table S1. Comparison of the study patients with and without DRF

|  | **DRF (+)**  **(n = 134)** | | **DRF (-)**  **(n = 205)** | | ***P* value** |
| --- | --- | --- | --- | --- | --- |
| Age, y (IQR) | 69 | (59−78) | 72 | (61−79) | 0.40 |
| Male, n (%) | 86 | (64.2) | 105 | (55.0) | **0.02** |
| **Site of infection, n** (%) |  |  |  |  |  |
| Central nervous system | 0 | (0.0) | 1 | (0.5) | 1.00 |
| Thoracic, pneumonia | 31 | (23.1) | 37 | (18.1) | 0.25 |
| Abdominal | 63 | (47.0) | 114 | (55.6) | 0.12 |
| Neck | 4 | (3.0) | 9 | (4.4) | 0.58 |
| Soft tissue | 10 | (7.5) | 8 | (3.9) | 0.15 |
| Urinary tract | 2 | (1.5) | 17 | (8.3) | **0.01** |
| CRBSI | 2 | (1.5) | 2 | (1.0) | 0.65 |
| Others | 23 | (17.2) | 17 | (8.3) | **0.01** |
| **Nephrotoxic agents, n (%)** |  |  |  |  |  |
| Aminoglycoside | 3 | (2.2) | 1 | (0.5) | 0.31 |
| Vancomycin | 51 | (38.1) | 51 | (24.9) | **0.01** |
| **Comorbidities, n** (%) |  |  |  |  |  |
| Ischemic heart disease | 16 | (11.9) | 22 | (10.7) | 0.73 |
| Chronic kidney disease | 32 | (23.9) | 24 | (11.7) | **<0.01** |
| Premorbid Cr (mg/dL) (IQR) | 0.86 n=79 | (0.72−1.34) | 0.76  n=104 | (0.6−0.92) | **<0.01** |
| eGFR <30 (%) | 19 | (24.1) | 2 | (1.9) | **<0.01** |
| Diabetes mellitus | 43 | (32.1) | 55 | (26.8) | 0.33 |
| Immunosuppressants | 38 | (28.4) | 62 | (30.2) | 0.71 |
| APACHE II score (IQR) | 30 | (25−305 | 22 | (17−27) | **<0.01** |
| SOFA score (IQR) | 10 | (8−12) | 7 | (5−10) | **<0.01** |
| SOFA (Non renal SOFA) | 9 | (6−10) | 6 | (4−8) | **<0.01** |
| SOFA (Renal SOFA) | 2 | (1−3) | 1 | (0−1) | **<0.01** |
| DIC, n (%) | 90 | (69.2) | 103 | (50.7) | **<0.01** |
| Septic shock, n (%) | 80 | (59.7) | 96 | (46.8) | **0.02** |
| Administration of CM | 45 | (33.6) | 87 | (42.4) | 0.10 |
| AKI stage on admission, n (%) |  |  |  |  | **<0.01** |
| Stage 1 | 30 | (25.4) | 88 | (42.9) |  |
| Stage 2 | 17 | (12.7) | 69 | (33.7) |  |
| Stage 3 | 87 | (64.9) | 48 | (23.4) |  |
| Cr on admission (mg/dL) (IQR) | 2.38 | (1.44−3.67) | 1.44 | (1.09−1.96) | **<0.01** |

Abbreviations: *CRBSI* catheter-related blood stream infection, *APACHE II score* acute physiology and chronic health evaluation II score, *SOFA score* Sequential Organ Failure Assessment score, *DIC* disseminated intravascular coagulation, *AKI* acute kidney injury, *Cr* creatinine, *IQR* interquartile range

Bold *P* values represent significant differences between with and without DRF
